# Rural landscape dynamics over time and its consequences for habitat preference patterns of the grey partridge *Perdix perdix*

**DOI:** 10.1371/journal.pone.0255483

**Published:** 2021-08-19

**Authors:** Sabine Marlene Hille, Eva Maria Schöll, Stéphanie Schai-Braun

**Affiliations:** University of Natural Resources and Life Sciences, Vienna, Austria; Sichuan University, CHINA

## Abstract

Intensification of agricultural practices has drastically shaped farmland landscapes and generally caused a decline in spatial and temporal heterogeneity, thus leading to changes in habitat quality and food resources and a decline for most farmland birds Europe-wide. The relationship between complex landscape changes and habitat preferences of animals still remains poorly understood. Particularly, temporal and spatial changes in diversity may affect not only habitat choice but also population sizes. To answer that question, we have looked into a severely declining typical farmland bird species, the grey partridge *Perdix perdix* in a diverse farmland landscape near Vienna to investigate the specific habitat preferences in respect to the change of agricultural landscape over two decades and geographic scales. Using a dataset collected over 7.64 km² and between 2001 and 2017 around Vienna, we calculated Chesson’s electivity index to study the partridge’s change of habitat selection over time on two scales and between winter and spring in 2017. Although the farmland landscape underwent an ongoing diversification over the two decades, the grey partridges declined in numbers and shifted habitat use to less diverse habitats. During covey period in winter, partridges preferred also human infrastructure reservoirs such as roads and used more diverse areas with smaller fields than during breeding where they selected harvested fields but surprisingly, avoided hedges, fallow land and greening. Known as best partridge habitats, those structures when inappropriately managed might rather function as predator reservoirs. The avoidance behaviour may further be a consequence of increasing landscape structuring and edge effects by civilisation constructions. Besides, the loss in size and quality of partridge farmland is altered by crop choice and pesticides reducing plant and insect food. With declining breeding pairs, the grey partridge does not seem to adjust to these unsustainable landscape changes and farmland practices.

## Introduction

Evidence has been numerous that since the 1950s farmland biodiversity decreases worldwide due to on-going changes in agricultural practices [1–3]. Agricultural intensification and specialization have been reshaping and simplifying agricultural landscapes leading to a loss of habitat and spatial and temporal heterogeneity [4]. Accordingly, degradation of habitat and food resources have been resulting in a severe reduction in biodiversity [1,3,5–7] and by more than 50% of the EU farmland bird indicator since 1980 [7–9]. Although agricultural land is covering about 40% of the terrestrial earth surface, so far only single studies have been assessing land-cover changes over time (see in [10]) or related them to changes in habitat choice of farmland birds (but see [11,12]).

Interestingly, in Europe, in large-scale intensive farmland the area of infrastructure such as roads as well as hedgerows and woodlands have mainly remained stable over the years [12], while crops have changed annually due to crop rotation [13]. In contrast, in less intensive agricultural areas e.g., in the Alpine regions, indicating less quality soil meadows or open habitats (e.g., set asides) change to higher succession stages (e.g., bush land or forests) because farmers lose financial revenues for the sustenance of such structures. Another reason is that land adds to the farmers income when treated as realty housing buildings or other infrastructure [14]. The latter may be more pronounced near housing development particularly in Alpine valleys and in close vicinity to cities, where land speculation highly affects land sells.

Most of the above-mentioned European studies focused on habitat choice in birds in intensive farmlands where infrastructure does not seem to change [12]. As a result, there is little evidence on the association of habitat preferences with the change of landscape heterogeneity over time and space caused not only by augmenting crop area size but also by increasing building and infrastructure. Thus, heterogeneity becomes a different meaning when concerning the habitat preferences of grey partridges. Worldwide more than 50% of all people lives in cities, with increasing tendency, therefore the habitat loss around cities is of severe present and future concern. Vienna is the most fast-growing big city in Europe with an increase in human population of 13% during the last decade [15]. The only possible expanding direction of Austria’s capital city is into rural eastern and southern areas where farmland vanishes due to buildings [15,16].

The grey partridge *Perdix perdix* (family: Phasianidae, Order: Galliformes) is a characteristic steppe—open land breeding bird that had been common in most farmlands in the 1950s [14–16] but drastically experienced a steep and wide-spread decline throughout Europe. In most countries, populations harbour today less than 10% of their pre-war numbers [17]. These declines were not linear in all countries because most losses in numbers happened in the last two to three decades. In Germany, numbers dropped since 1992 by 89% and between 2004 and 2016 by 50% (Dachverband Deutscher Avifaunisten, DDA). In France, the distribution between 1989 and 2015 went down to remaining 23% [18]. In the UK, the loss exhibited about 92% between 1970 and 2015 and, as a consequence, the bird was included on the Red List [19]. In Austria, the short-time decline between 2011 and 2016 exhibited 47%, but the long-time trend showed a drop by 82% between 1998 and 2016 [20]. The falling by the wayside of the partridge illustrates an indicator for drastic Europe-wide landscape change and habitat loss due to agricultural intensification. Potts and Aebischer [21] and Aebischer and Ewald [22] attributed the massive declines to 1) the loss of breeding habitat, 2) the decrease in availability of insects for chick food and 3) the concentration of grey partridges and predators in remaining few habitats.

The needs and habitat preferences of grey partridges are well studied. As a steppe bird, they avoid forests and trees [23], and buildings [24] because they often harbour predators. Breeding partridges search for cover in cereals [25,26], grass or rape that is not too dense [25,27]. Partridges feed mainly on plant seeds but during breeding they need arthropods in order to produce eggs, rear young and promote moult. Further, they need structural cover as a protection from predation [28]. Partridges profit from a mosaic of wild-flower strips that are ideally mowed in a rotating order [29]. Survival of partridge chicks and adults is lower in regions with a simplified agricultural landscape structure, e.g., large fields, pesticide use and a lack of permanent cover [30–32]. The birds can often be found along linear structures such as tracks, field margins, stripes along paths and hedges to find food and shelter [21–25,33]. However, breeding success is not always high in and besides these linear structures [26]. Particularly when the structures for hiding are small and additional predator access from guiding linear structures is given. Thus, hiding structures need to be higher and larger in habitats with high predation pressure [28]. Gottschalk and Beeke [29] showed that small fallow fields exhibited a high predation with 75% of nest lost before hatch, of these, 80% were predated by mammals and 20% by aerial predators. Blooming stripes and fallow land need to be at least 20 by 20 meters, frequent and connected to provide a hen to guide the chicks without losing the cover [34]. There seems a causal connection between field size and breeding success, respective predation rate [29,35].

The availability of hedges as good breeding habitat including dead grass providing concealment of nests is controversially discussed [22,30,36,37] but see [38–40]. Aebischer & Potts [41] claimed that partridges need at least 4 km hedges to maintain brood success in British intensive farmlands. But hedges if not cultivated can turn into treelines with no adequate herbal hiding below and nice predator perches above [39]. Thus, the different findings on breeding success in and near hedges including the dynamic quality of a hedge as partridge shelter over time require further studying.

In most of the studies on habitat preference in grey partridge, the effects of landscape features have been studied independently from each other. Consequently, there is still little known on the relative contributions of single landscape parameters for the partridge’s habitat preference in farmland landscape in space and time (but see [12,24,40]). Even less is known about potential changes in patterns of habitat preference over time in highly dynamic agricultural landscapes, particularly close to cities.

The goal of this study was to investigate between-year and within-year trends in grey partridge habitat preferences in a peri-urban farmland landscape. Our hypotheses were: (1) grey partridge populations in the vicinity of the city Vienna are declining following the Europe-wide trend; (2) grey partridges’ habitat preferences are influenced by hedges of different quality; (3) grey partridges’ habitat preferences change according to their social structure and reproductive state; (4) habitat preferences of grey partridges change in compliance with the highly dynamic agricultural landscapes close to cities; and (5) grey partridges’ habitat preferences alter in accordance with available diversity and infrastructure in agricultural landscapes near cities.

To study these goals, we analysed data covering three study periods within 17 years from grey partridge counts and landscape mapping in the agricultural landscapes near Vienna at two spatial scales, and investigated the relative importance of single habitat structures, field size and diversity on this bird’s habitat preference.

## Materials and methods

Grey partridges were monitored in the years 2000–2002 [42], 2008–2012 [43] and in the year 2017 (within our study) in arable habitats close to Vienna (see also S1 Fig). Within the two previous studies, grey partridges in Vienna district were counted within squares consisting of nearly all habitat that was assumed to be suitable for grey partridges (see [42] and [43] for details). Each single square covered a sixth of a geographical minute (field site length of 618 m) and birds were counted along transects adapted to the local conditions. Since field use, time of year and day, disturbances, wind–and other weather conditions all affect the behaviour of partridges, the field worker needed to adapt to this variation. Thus, transect position and length within the squares might have changed between 2002 and 2012.

In the year 2017, we proceeded a resampling of the squares and were able to select a practicable number of 20 squares (S1 Fig and S1 Table) covering most of the partridge habitat in an area of 7.64 km² on the basis of two main criteria: i) grey partridge occurrence was detected at least within one of the two previous studies within the square [42,43]; ii) at least 10% of the total square surface area was still suitable for grey partridges in the year 2017. Both criteria had to be fulfilled.

### Mapping grey partridges

Grey partridge occurrence was assessed within two field trips (winter, spring 2017) which were performed by three trained field observers. A first field trip was conducted in winter during daytime (2^nd^ - 16^th^ February 2017, Fig 3), when agricultural landscapes were covered by snow and individual partridges, pairs and coveys (> 2 individuals) were easily detected. Since field paths did not allow for detecting grey partridges within the whole square, additional pathways were determined by foot according to the actual habitat structure to cover the entire square. Thus, the observers walked through the squares at a steady, slow pace and recorded the location of grey partridges (transect count). Thereby, we assume that detection probability did not differ between habitat types. We used one long transect per square to easily cover the entire area. We did not want to change the transect count method that had been used in the previous years, thus, we did not use replicate counts.

The second field trip was made in spring during breeding season (9^th^ March - 10^th^ April 2017, Fig 2). As performed in the two previous studies by Wichmann and Teufelbauer [42] in the years 2000–2002 and Sabathy [43] in the years 2008–2012, we surveyed grey partridges during breeding season by using a vehicle to drive to survey points distributed within the squares (point count). Number and position of survey points was adapted to terrain and vegetation cover of each square (see also [43]). At each survey point, we visually observed squares and listened for spontaneously calling males. In addition, we used playbacks for one minute and counted the answering males during the next two minutes after playback (see [42,43]). Vocal activity was found to be relatively constant between mid-March and mid-April [44] and males have their highest vocal activity during sunrise [36] and sunset [44]. Therefore, grey partridges were counted either in the early morning (starting 90 minutes before sunrise, see also [36]), or in the evening (starting 90 minutes before sunset, see also [44]) lasting for a total period of 3 hours. We are aware that using playback calls may increase detection probability of grey partridges by 1.3 to 1.6 times [45,46], but in order to achieve comparable data with the previous studies of Wichmann and Teufelbauer [42] and Sabathy [43], we used this field protocol.

The location and movement of each grey partridge (individual or covey) counted in winter and spring 2017 was mapped in a field map. In addition, when more than one grey partridge was detected within a time, we added remarks to avoid false double counts.

### Mapping habitat characteristics

To determine habitat preference, habitat availability and use by partridges have to be ascertained. Availability and use were determined by way of habitat mapping. The habitat mapping was conducted in 3.14 ha circular plots (100 m radius) around each grey partridge location (habitat use). The area of 3.14 ha around each grey partridge location should cover the home range size of grey partridges during breeding season [34,47]. In squares without detection of grey partridges, the centre of the squares was used to set reference circular plots with equal size (habitat availability). Habitat types found within the circular plots were then determined at a rough and fine scale using a geographical information system (ArcGIS, Esri).

### Mapping land cover types within the agricultural landscape on a rough scale

We digitally analysed land cover of the 20 squares on a rough scale by using open government data (annual landcover maps and aerial photographs; Stadt Wien - https://data.wien.gv.at, S2 Table). Since Wichmann and Teufelbauer [42] collected grey partridge occurrence data from the years 2000–2002, we used annual land cover maps and aerial photographs from the year 2001 to determine land cover types. Sabathy [43] counted grey partridges in the east and north of Vienna in the years 2008–2010, and in the south of Vienna in the years 2011–2012. Thus, we used annual land cover maps from the year 2009, and aerial photographs from the years 2008 and 2012 for the different sites, respectively. In addition, land cover maps and aerial photographs of the year 2016 were used for our own data on grey partridge occurrence, since we assume that these land cover classes were still predominant in early 2017, when we conducted our field work.

The different landscape types (e.g., arable lands, meadows, vineyards, forests, hedgerows and water bodies) and infrastructures (e.g., roads, field paths, settlements, areas under construction, parks, power poles and windmills) were quantified, as they could be distinguished using the aerial photos. The sum of all habitat types found in the circular plot around each grey partridge location comprised the habitat use of each individual grey partridge. The sum of all habitat types in the circular plot around each control point was averaged over all squares and, thus, comprised the total habitat available to all grey partridges. Distances to the nearest infrastructure for each observed partridge or control point in the year 2017 were calculated in the geographical information system using the proximity analysis tool.

### Mapping habitat characteristics on a fine scale in 2017

Each individual grey partridge location, as well as the centre of those squares without detection of grey partridges were visited again later on to record fine scaled habitat parameters (see also [42], S3 Table). Locations were visited within the same day when counting of grey partridges had been conducted in the early morning, or the day after when grey partridge occurrence had been assessed in the evening. Within the circular plots, we collected data on the use of agricultural areas and set-asides (e.g., winter grain, harvested field, without crop, meadow, fallow land/green manures) and all additional habitat structures, which might be also used by grey partridges (e.g., field paths, gravel walks, forest islands). We also obtained data on height and width of hedgerows, small tree islands, and density of understory.

To discriminate hedges that are of particular use for partridges, providing shelter and food from those overgrown hedges, not suitable habitat but rather a perch for predators, we created the variable “super hedge”. We used the parameters height (>4m) and width of hedge (>2m) and availability of a dense understory (herbal vegetation cover below ground >70%) to identify super hedges. We identified hedges and super hedge characteristics as precisely as possible on the maps, since the aerial maps do not really reflect height and ground cover, we at random identified and mapped in 30% of available hedges these variables in the field. But only too few hedges fit our expectations as being low, bushy, thick and with herbal cover as partridge quality hedges. Finally, all hedges remained within the model because they were more or less all rather tall, overgrown bush or tree-lines with nearly no ground cover, no good partridge habitat.

Available habitat types were not averaged over all squares because averaging proved to include habitat types not available in the individual squares and causing thus too many avoidances.

To assess diversity, we calculated the number and sizes of areas available in a square. Thereby, we defined a plot covered by a landscapes type as an area.

### Data analysis

All data analyses were carried out using the software R 3.6.3 [48]. Habitat preferences were measured by using Chesson’s Electivity Index ε [49], an index based on Manly’s alpha [50].

$$\alpha_{i}=\frac{r_{i}}{n_{i}}\times \frac{1}{\sum_{j=1}^{m}\left(\frac{r_{j}}{n_{j}}\right)}$$ (1)

(1): Manly’s alpha selection index (α) [50]. α_i_ = Manly’s selection index for habitat type i; r_i_, r_j_ = proportions of habitat types i and j in the partridge’s habitat (i and j = 1, 2, 3, …, m); n_i_, n_j_ = proportions of habitat types i and j available; m = number of potential habitat types.

$$\varepsilon_{i}=\frac{m\alpha_{i}-1}{(m-2)\alpha_{i}+1}$$ (2)

(2) Electivity index ε [49]. m = number of potential habitat types, α_i_ = Manly’s selection index for habitat type i.

Chesson’s Electivity Index has the advantage that individual habitat preferences are comparable for a varying number of habitat types available to different individuals [51]. The Chesson’s Electivity Index ranges between -1 and +1; negative values are related to negative selection (avoidance, -), whereas positive values show positive selection (preference, +). The Chesson’s Electivity Indices were calculated for each square where grey partridges were mapped, for each individual grey partridge, and for each grey partridge territory. The Indices were calculated based on different habitat mapping: the annual land cover map (years 2001, 2009 and 2017) and the fine scale habitat map (year 2017) with and without differentiation in super hedges. The reliability of Chesson’s Electivity Index was tested by using the bootstrap method [52] using the package “boot” [53]. The original ε_i_ values (ε_i_ = Chesson’s Electivity Index for the habitat type i) were resampled 1500 times with replacement and an accelerated bootstrap confidence interval was calculated. The accelerated bootstrap adjusted the confidence interval for bias and skewness [54]. If the lower and upper 95% CI featured different algebraic signs, the selection for the respective habitat type were not significant (n.s.). ε_i_ values for habitat types were only bootstrapped if they were calculated for 7 or more squares with grey partridge presence/grey partridge individuals/grey partridge territories, as smaller sample sizes provide unreliable results. Shannon-Wiener-Indices were calculated using the package “vegan” [55].

### Statistical analysis

Generalized linear mixed-effects models were fitted using the package lme4 [56]. Firstly, the response variable Shannon-Wiener-Index was investigated with a model including the covariate type (used vs available). Secondly, two models including the covariate year (2001 vs 2009 vs 2017) scrutinised either the response variable number of grey partridges or number of grey partridge territories. Thirdly, several models including the covariate year (2001 vs 2009 vs 2017) scrutinised the response variables average number of areas and average area size per square for used and available habitat, and infrastructure. Fourthly, several models including the covariate type of habitat (used vs. available) or season (winter vs. spring) scrutinised the response variable distance to the nearest infrastructure in the year 2017. All models included the variable “square” as random factor in order to account for the repeated measurements collected from the different squares. As the program R does not directly provide *p*-values for such models, the *p*-values were extracted by likelihood ratio tests [57]. Residuals of the models were checked for normal distribution by QQ-plots and histograms. The homogeneity of variances and goodness of fit were examined by plotting residuals versus fitted values [57]. The relationship between total infrastructural area and year was investigated using the Pearson correlation coefficient.

## Results

### Distribution and territory numbers

**Fig 1:**
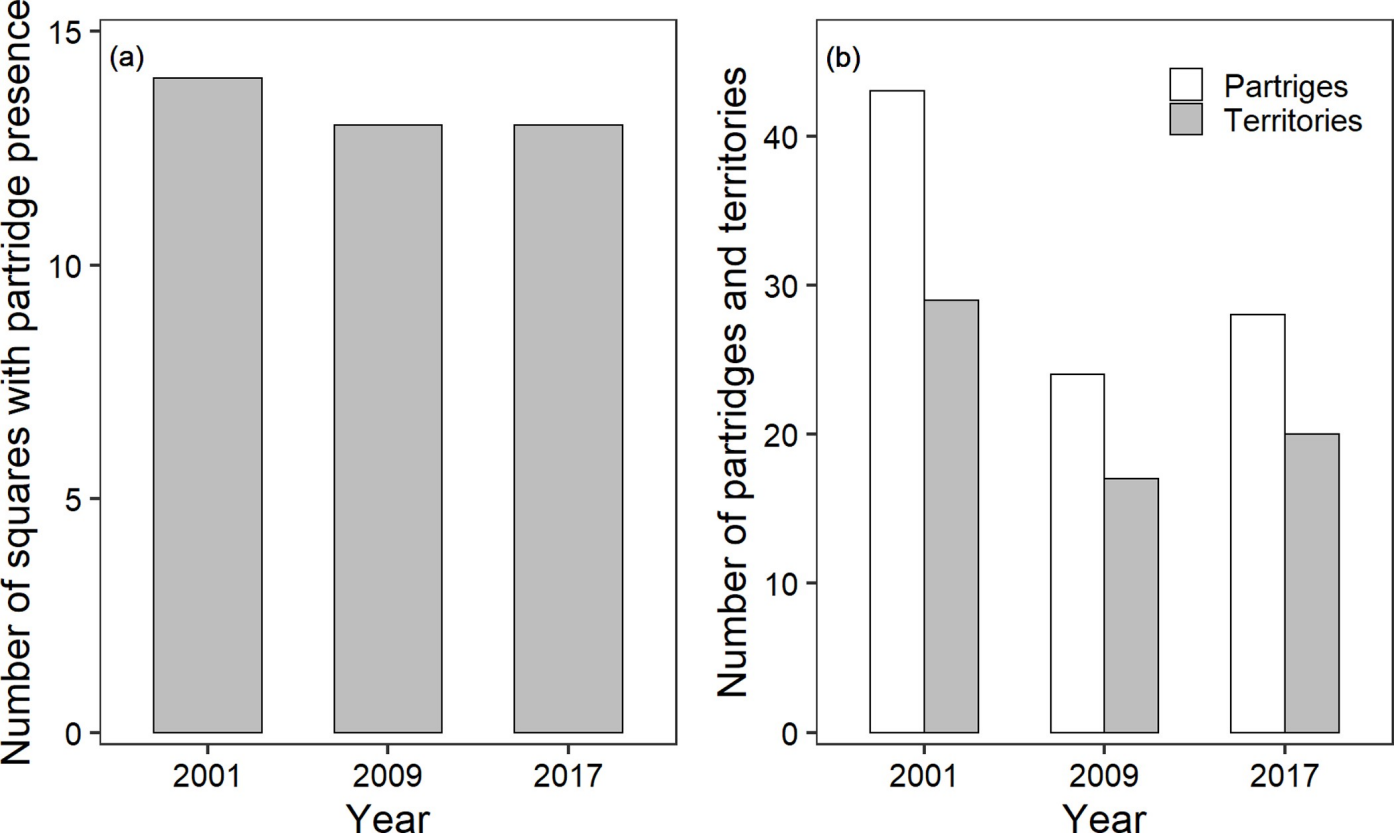
Habitat occupation of grey partridges in Vienna district. Habitat occupation of grey partridges in Vienna district for the years 2001, 2009 and 2017 differing between (a) numbers of squares with grey partridge presence and (b) numbers of grey partridge individuals (white) and numbers of territories (grey).

**Fig 2:**
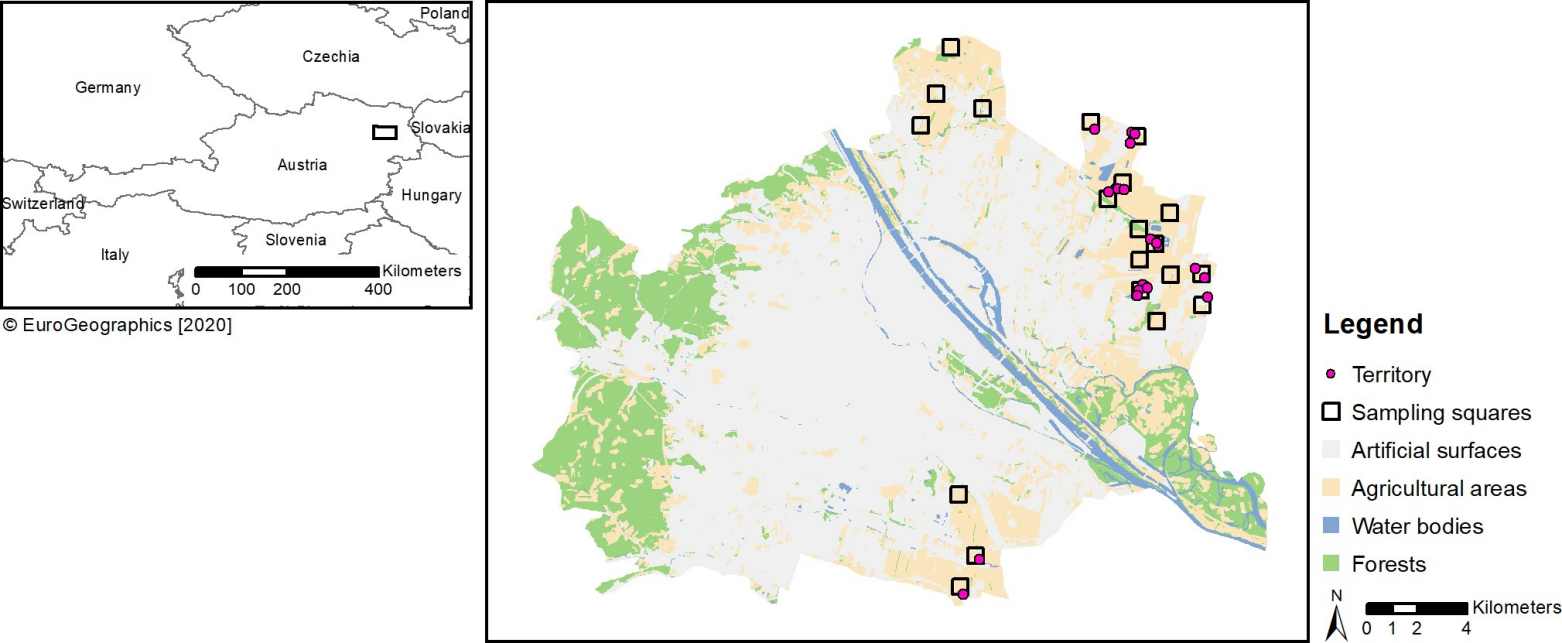
Spatial distribution of territorial pairs in spring 2017. Artificial surfaces include settlements and infrastructure (e.g., roads). Source Realnutzungskartierung 2016: Stadt Wien - https://data.wien.gv.at.

**Fig 3:**
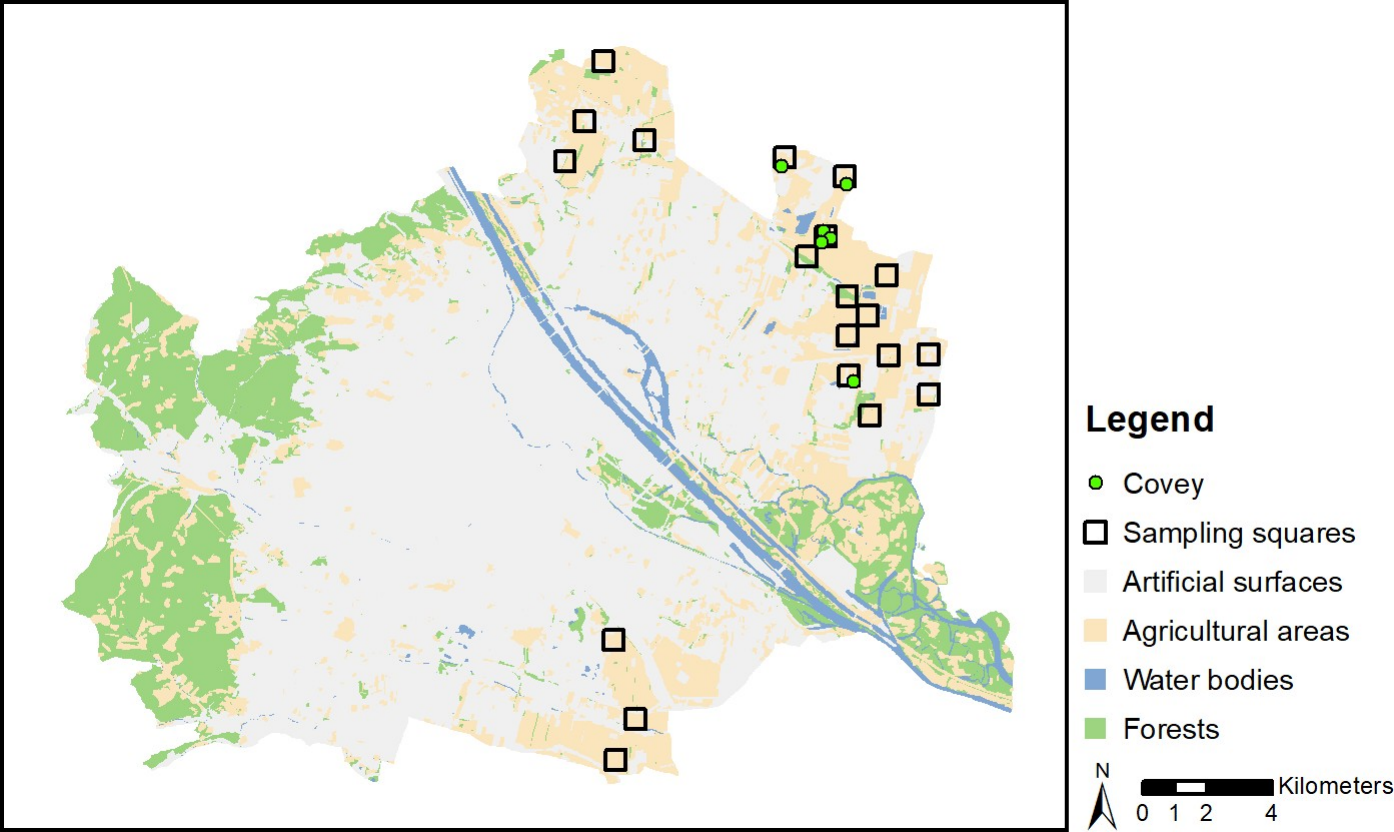
Spatial distribution of coveys (> 2 individuals) in winter 2017. Covey size varied between 3 and 15 individuals (mean = 7.2 individuals). Artificial surfaces include settlements and infrastructure (e.g., roads). Source Realnutzungskartierung: Stadt Wien - https://data.wien.gv.at.

The number of squares occupied by partridges remained the same over the years (X² = 0.151; p = 0.927; Fig 1A) but estimation of the numbers of individuals and territories revealed a visible decline over the years. Since the lowest population estimate appeared in 2009, the overall change in bird numbers (X² = 3.501; p = 0.173; Fig 1B) and territories (X² = 2.946; p = 0.229) over the years was not significant. To compare spatial distribution of grey partridges in winter and spring 2017 see Figs 2 and 3.

### Available habitat and the partridge’s habitat use

In the available habitat for grey partridges, habitat diversity in terms of average area size (X² = 12.042; p < 0.001) and number of areas (X² = 7.569; p = 0.006) per square increased significantly over the years 2001, 2009 and 2017 (Table 1). The habitat diversity increase was highest from the year 2009 to 2017 in having almost the threefold number of areas and one-fifth of the area size per square in 2017 compared to 2009. In the squares, present infrastructure (i.e., roads, field paths, settlements, parks, windmills, power poles, sites under construction) increased considerably over the years 2001, 2009 and 2017 as the average number of infrastructural areas (X² = 3.169; p = 0.075; Table 2) increased significantly, whereas the average infrastructural area sizes (X² = 2.165; p = 0.141) remained constant. In line with this, correlation coefficient demonstrates a strong increase in total infrastructural area during the time period (ρ = 0.942).

**Table 1:** Change of habitat diversity for the years 2001, 2009, and 2017.

a)	Habitat availability	Habitat use		
Year	Average nr of areas per square	Average nr of areas per square with partridge presence	Average nr of areas per partridge	Average nr of areas per territory
2001	3.6	4.5	4.7	4.5
2009	7.9	5.9	5.8	5.9
2017	21.1	8.7	8.0	8.7
b)	Habitat availability	Habitat use		
Year	Average area size per square (ha)	Average area size per square with partridge presence (ha)	Average area size per partridge (ha)	Average area size per territory (ha)
2001	1.3	1.3	1.3	1.3
2009	1.1	1.0	1.0	1.0
2017	0.2	0.8	0.8	0.8

Change of habitat diversity for the years 2001, 2009, and 2017 characterized by (a) the average number of areas and (b) the average area size. Habitat diversity is measured for available habitats to grey partridges and for used habitats by grey partridges.

**Table 2:** Change of present infrastructure for the years 2001, 2009, and 2017.

Year	Average nr of infrastructural areas per square	Average infrastructural area size per square (ha)	Total infrastructural area (ha)
2001	1.3	0.1	106.9
2009	5.4	0.3	117.5
2017	8.7	0.1	152.9

Change of present infrastructure (i.e., roads, field paths, settlements, parks, windmills, power poles, sites under construction) in the squares for the years 2001, 2009, and 2017.

In the habitat used by grey partridges, habitat diversity augmented also over the years although only significantly regarding the number of areas and not regarding the area sizes. This was true for the squares with partridge presence (average area size: X² = 2.576; p = 0.109; average number of areas: X² = 8.402; p = 0.004; Table 1), as well per partridge (average area size: X² = 1.524; p = 0.217; average number of areas: X² = 8.881; p = 0.003) and territory (average area size: X² = 2.576; p = 0.109; average number of areas: X² = 8.402; p = 0.004). However, habitat diversity was not as high in used habitats as in available ones. Hence, grey partridge did not choose habitats especially rich in habitat diversity. This was particularly pronounced for the year 2017. The distances to infrastructure were not significantly different between partridges and control points both in winter and in spring of the year 2017 (p > 0.05). However, partridges were found significantly nearer to infrastructure in winter (median: 22.19 ± 7.52 SE) than in spring 2017 (median: 112.85 ± 15.69 SE; X² = 21.022; p < 0.0001).

**Fig 4:**
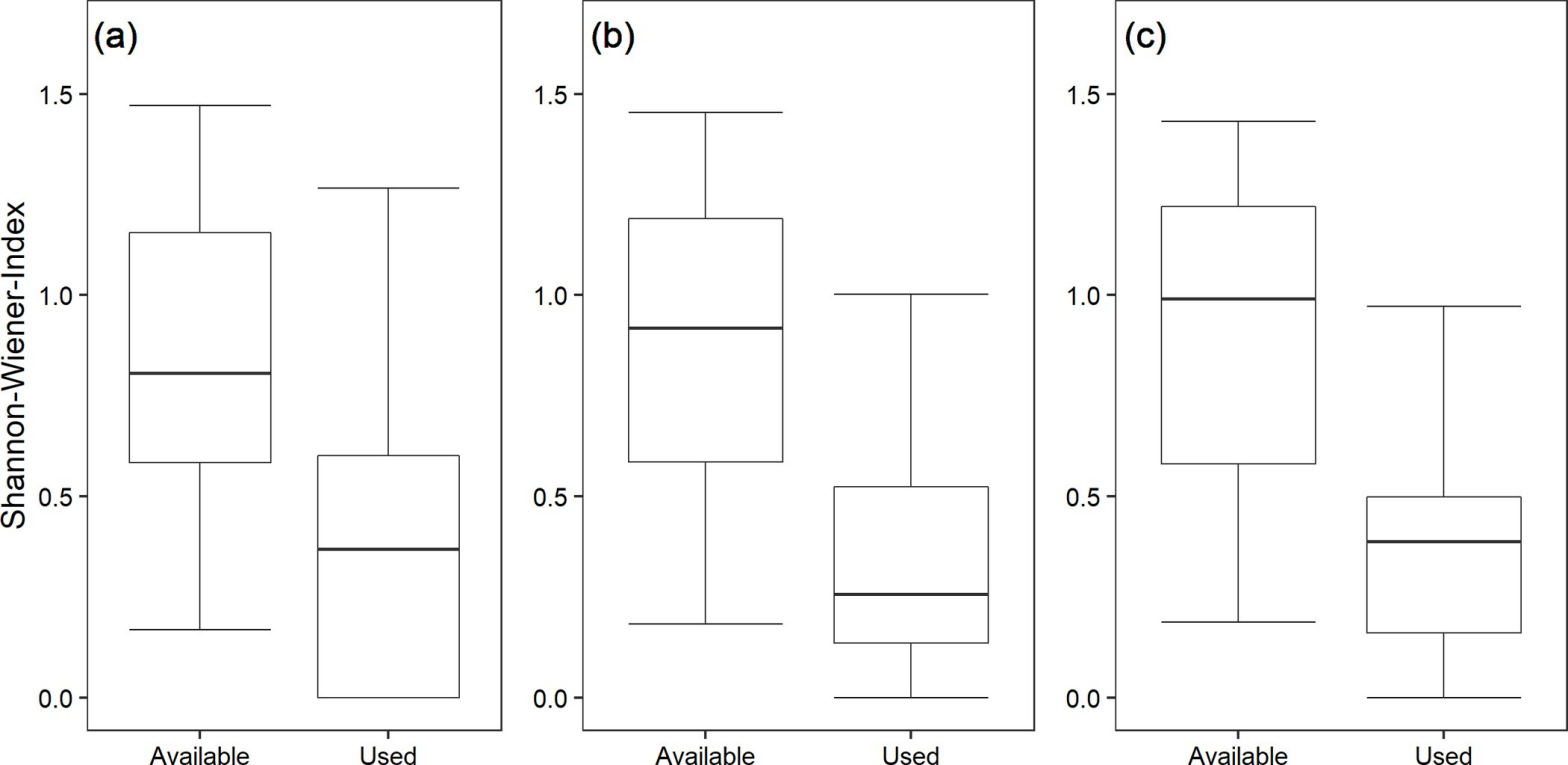
Habitat diversity available to and used by the grey partridge. Habitat diversity available to and used by the grey partridge in Vienna district for the years (a) 2001, (b) 2009, and (c) 2017. Habitat diversity was measured by the Shannon-Wiener-Index (medians with 25^th^/75^th^ and 10^th^/90^th^ percentiles) using the annual land cover map. See text for statistical details.

Shannon-Wiener-Indices proved to be significantly smaller for used habitats than for available habitats for the years 2001, 2009, and 2017 (2001: X² = 22.56; p < 0.001; 2009: X² = 27.92; p < 0.001; 2017: X² = 25.99; p < 0.001; Fig 4). Thus, grey partridges selected their habitat for low habitat diversity in all years (Table 1 and Fig 4).

### Habitat selection in spring at the rough habitat scale

**Fig 5:**
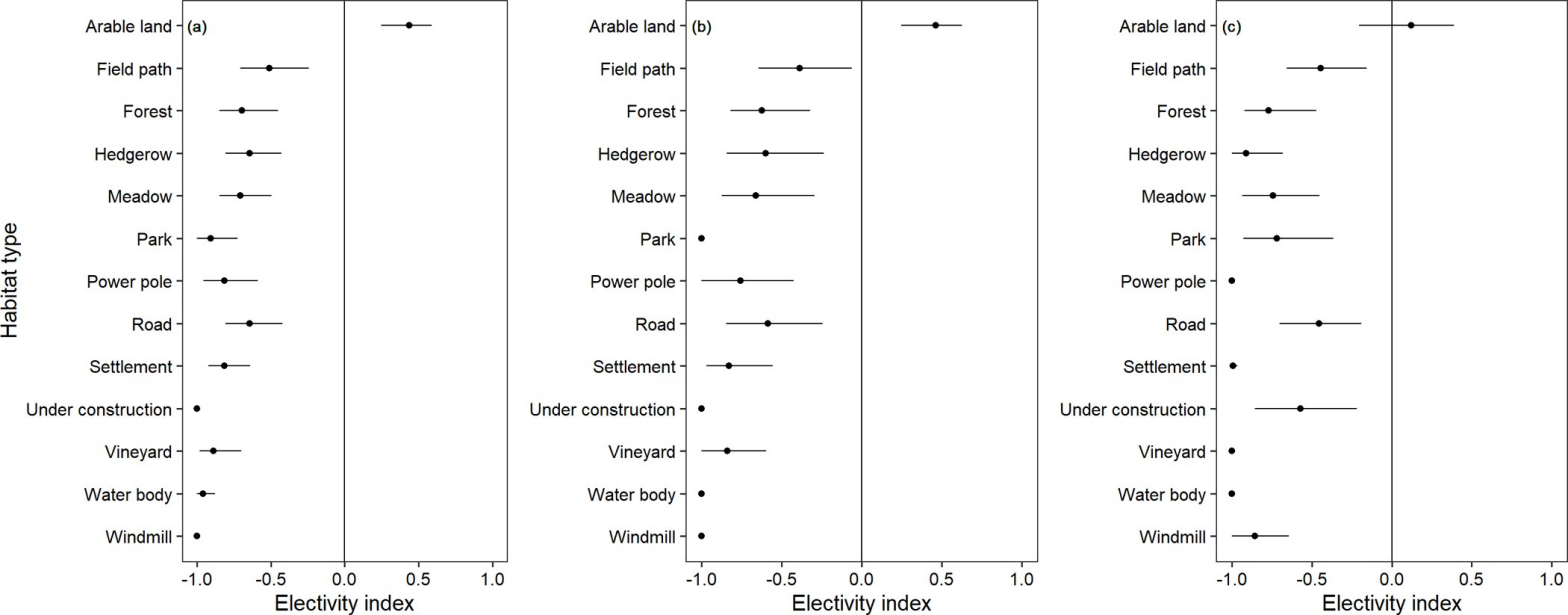
Chesson’s electivity indices for habitat types of the grey partridge in the years 2001, 2009 and 2017. Chesson’s electivity indices and their distributions of 1500 bootstrap resamples (mean and 95% confidence interval) for habitat types of grey partridges based on the annual land cover map. Grey partridge individuals were mapped in Vienna district, Austria, during (a) 2001 (n = 43), (b) 2009 (n = 24), and (c) 2017 (n = 28). Not significant results cross the vertical line at zero. See text for statistical details.

At the rough habitat scale (Realnutzungskartierung), partridges preferred arable land in 2001 and 2009, whereas the same habitat type was neutrally selected in the year 2017 (Fig 5).

In the years 2001, 2009 and 2017, grey partridges avoided significantly the habitat types field path, forest, hedgerow, meadow, park, power pole, road, settlement, under construction, vineyard, water body and windmill at the rough habitat scale.

### Change of distribution and habitat use within the year 2017

In the habitat used by grey partridges, habitat diversity in terms of average area size and number of areas per square decreased from winter to spring census in the year 2017 (Table 3). This was true for the squares with partridge presence, as well per partridge. Hence, the birds chose habitats especially rich in habitat diversity during the covey period, whereas during breeding season habitats with lower diversity were used.

**Table 3:** Change of habitat diversity for winter and spring censuses in 2017.

a)		
Year and census	Average nr of areas per square with partridge presence	Average nr of areas per partridge
winter 2017	13.5	14.9
spring 2017	8.7	8.0
b)		
Year and census	Average area size per square with partridge presence (ha)	Average area size per partridge (ha)
winter 2017	3.6	3.1
spring 2017	7.9	8.5

Change of habitat diversity for the winter and spring censuses in the year 2017 characterized by (a) the average number of areas and (b) the average area size. Habitat diversity is measured for used habitats by grey partridges.

### Habitat selection in winter 2017 at the rough habitat scale

**Fig 6:**
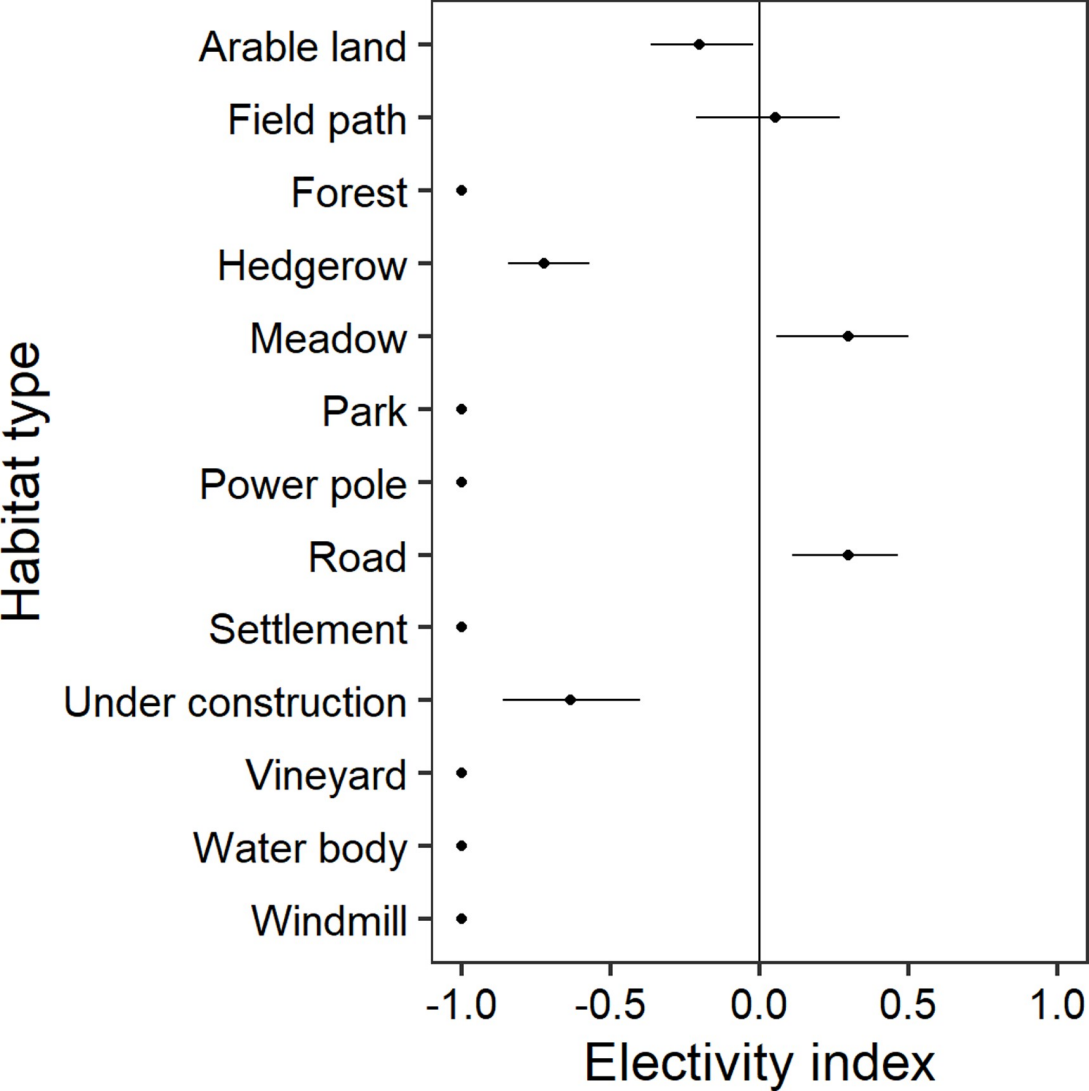
Chesson’s electivity indices for habitat types of the grey partridge during winter 2017. Chesson’s electivity indices and their distributions of 1500 bootstrap resamples (mean and 95% confidence interval) for habitat types of grey partridges in winter based on the annual land cover map. Grey partridge individuals (n = 43) were mapped in Vienna district, Austria, in the year 2017. Not significant results cross the vertical line at zero. See text for statistical details.

At the rough habitat scale, grey partridges preferred significantly meadows and roads in winter, whereas field paths were neutrally selected (Fig 6). The habitat types arable land, forest, hedgerow, park, power pole, settlement, under construction, vineyard, water body and windmill were avoided significantly by grey partridges.

### Habitat selection in spring 2017 at the fine habitat scale

**Fig 7:**
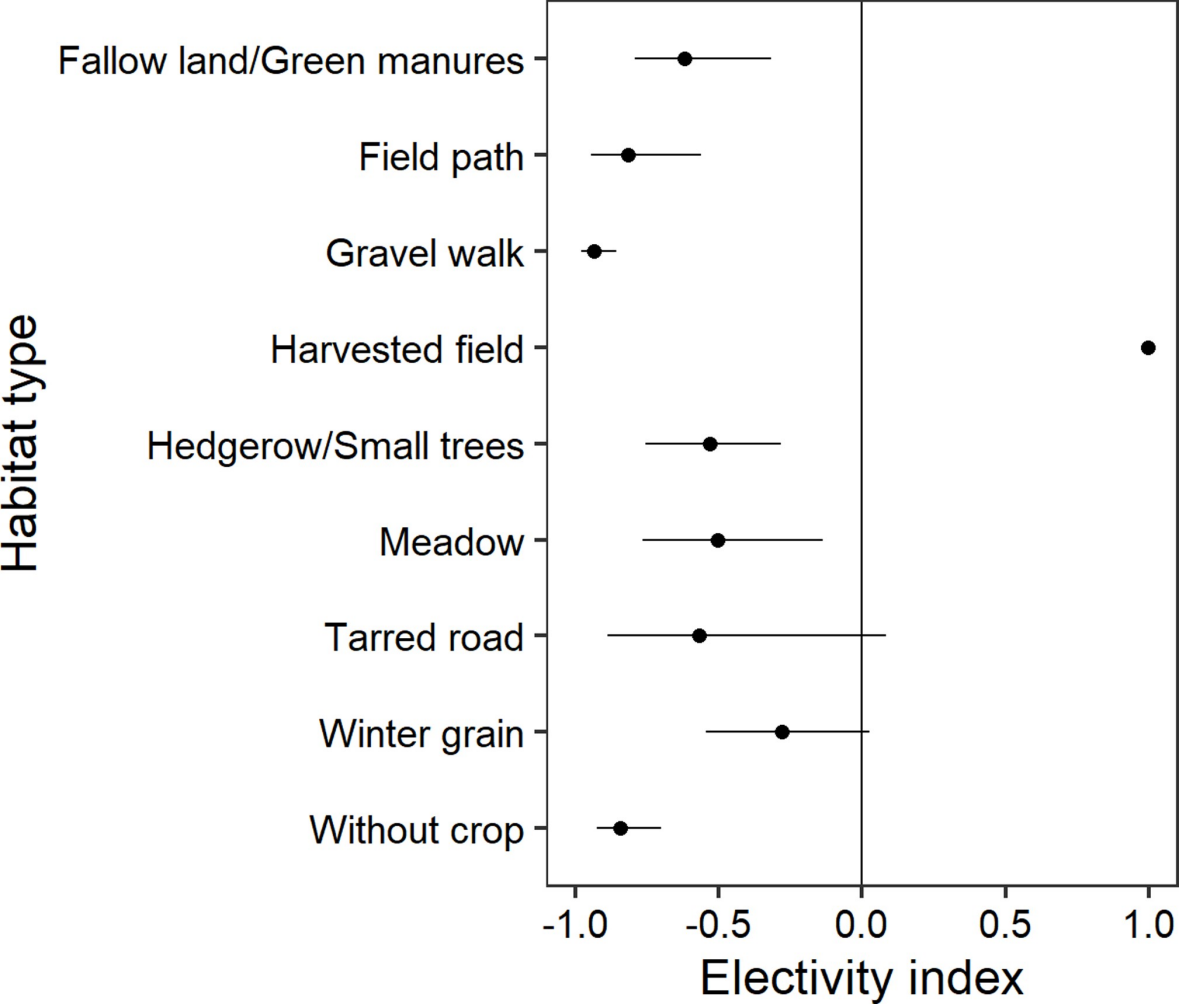
Chesson’s electivity indices for habitat types of the grey partridge during spring 2017. Chesson’s electivity indices and their distributions of 1500 bootstrap resamples (mean and 95% confidence interval) for habitat types of grey partridges in spring based on the fine scale habitat map. Grey partridge individuals (n = 68) were mapped in Vienna district, Austria, in the year 2017. Not significant results cross the vertical line at zero. See text for statistical details.

At the fine habitat scale, grey partridges preferred harvested fields in spring 2017 (Fig 7). The habitat types fallow land/green manures, field path, gravel walk, hedgerow/small tees, meadow, and without crop were avoided, whereas tarred road and winter grain were neutrally selected. Note that when including the habitat type “super hedge” in the fine scale habitat analysis, super hedge did not appear as selected habitat type by grey partridges as less than 7 squares with grey partridge presence/grey partridge individuals/grey partridge territories included super hedge in their habitat.

## Discussion

The mean size of agricultural fields in a landscape has been demonstrated by several international scientists to be one major driver of diversity and abundance of farmland biodiversity taxa including plants, arthropods, and vertebrates [34,58,59]. They concluded that small field sizes are of utter importance to halt and maybe even reverse the decline in biodiversity in landscapes dominated by cropping systems, which cover about 19% of the terrestrial land area [60]. In contrast, close to cities, a decrease of farmland patches is mostly caused by increasing elements of infrastructure (Table 2) but this generated landscape diversity does not necessarily reflect an increase of biodiversity. One key outcome of our study is an increasing farmland diversification over decades in Vienna’s agricultural sites and, above all, grey partridges shifted habitat preference not towards diverse habitats but, in contrast, they preferred in all years less diverse habitats, most pronounced in 2017 (Fig 4 and Table 1). Indeed, population size of grey partridges in Vienna has been decreasing, assuming that this type of diversity and fragmentation does not seem to maintain habitat quality.

We showed a farmland decline in Vienna district between 1997 until 2016 from 16.49% to 13.75%, representing a loss of 1136.31 ha over 20 years. That change parallels the increase in building and traffic infrastructure cover (Realnutzungskartierung, Statistik Austria 2017). When landscape diversity is increasing due to the rising of infrastructure, resulting further to a decrease in field or habitat size, the typical steppe bird firstly suffers direct habitat loss. Secondly, the connectivity of different fields providing enough food and shelter to guide the young is impaired and, thirdly, the bird loses the open, steppe-like good overview for predators.

Housing in Vienna is growing mainly into open Southern and Eastern farmland areas and former partridge habitat directly got lost. This development leads to fragmented or even isolated farmland patches that might be even rich in insects or herbal cover but if not connected and too close to frequented settlements and dog walking paths result in an avoidance of partridges. Although this farmland habitat may still be present, it is lost for partridge use, since they need at least 1–2 ha connected different-aged fallow land and flowering stripes between arable fields during breeding [34] to safely guide their young in cover. The main limiting factors for population numbers are the habitat quality and availability during incubating and guiding the young between May and mid-August [37,38].

Predation, connected to habitat fragmentation, further alters habitat quality and is a key factor for a decline in reproduction [29], particular in non-adequate habitats [35]. In partridge habitats that are easily accessible for predators (e.g., agricultural fields close to settlements, streets, forests, tree lines and small rest habitats), the chances increase that foxes, mustelids, or cats converge with grey partridges [29,37]. Bro et al. 2004 [61] demonstrated that small structures increase predation risk and patches in highly fragmented areas may act as ecological traps in which birds suffer from high predation. To maintain reproduction and survival of partridges, the habitat patches need to be firstly large enough that sensing the birds and meeting them by chance is widely reduced for a predator. Secondly, the habitat patches need to be in distance to predator harbouring islands and lines along they orientate and, thirdly, they need to be open and steppe like in order to oversee the area clearly. Any structures—even though they harbour seeds or insect food attractive for partridges—that maintain or can be used as orientation paths for predators or facilitate them to rest, negatively affect partridge numbers [32]. Predators are then even facilitated to get closer into the crop fields and, consequently, directly into partridge breeding habitat. Distances of partridges to woodlands and buildings during breeding are expected because of avoidance of these carnivore reservoirs [23,24,62]. Indeed, we found that in all years, partridges avoided these structures

Potts’ [37] review showed the importance of hedgerows as breeding sites in intensive British farmlands (but see [63,64]). Harmange et al. [12] asserted that in France the number of roads, tracks, and hedgerows have remained fairly stable over the last decades and hedgerows did not contribute much to their model on the probability of occurrence of partridges. When evaluating field types and structures, in particular hedges in their quality as good partridge habitat, we need to look not only into their abundance but also at their succession changes over time. In our study area, the majority of hedges fit not our expectations to be good quality partridge hedges with low height, bushy, thick and with herbal cover. Partridges avoided hedges in all years indicating, that with their age and mostly overgrown, they may not function as good habitats to find food and shelter. A hedge if not cultivated changes depending on the species composition over five to 10 years from small bushes with very adequate herbal cover and food for the birds to bare treelines that offer no sufficient ground cover and shelter but perches for birds of prey and facilitate ground predator movement. In Britain, it is shown that the most suitable nesting habitat for grey partridges exists in the hedges trimmed every other year [39]. Overgrown hedges may function as little woods, and woodlands harbouring predators have already been identified as the main driver of grey partridge distribution at local district scale [40].

To understand the relationship between complex landscape changes and habitat preferences of animals in particular, we need to identify heterogeneity and its temporal and spatial changes because they may affect not only habitat preferences but also population sizes. According to the decline of the Austrian farmland bird index [65] with the partridge accounting highest to the index, numbers of grey partridges dropped also in Vienna, representing the drastic declining European trends. In line with other European studies, population numbers in Vienna are fluctuating but decreasing [12] (Fig 1). Potts [37] affirmed that today most partridges worldwide live on arable land, a secondary artificial habitat. The birds live mainly in crops and grazing pastures, depending on small weed seeds and insect food during breeding. Our results indicate a preference for arable land between 2001 and 2009, but not in 2017 (Fig 5). Partridges exhibited in the last study years no preference for either habitat assuming they were using the habitat categories equally. Cereals is well known as the main breeding cover selected by grey partridges (69% of nests in cereals [12,25]), associated with high nesting success [26]. However, the authors demonstrated a response curve of nesting success to cereal cover that exhibits saturation, indicating a need for complementary habitats, for example, rape for food [27], mainly insect, seed and cover rich fields and stripes as described in Oppermann et al. [34]. Arable land gets less suitable if these structures are missing. In nowadays intensively managed farmlands, the key variable for good partridge habitat is diversification in open habitat, referring to a change or mosaic in crop types, meadow, blooming stripes, fallow with differing ages, young hedges and bushes, and several differing field margins but all placed in open farmland habitat, at least in a minimum size and in vicinity to each other but far from forest, roads [34,36,37,66] and settlements [12].

At a higher resolution, our analyses on habitat and within year variation illustrates partridges to switch from diverse areas in covey period to less diverse areas with larger fields in breeding time (Table 3). After winter, the pre-nesting dispersal is crucial to space nests and to become inconspicuous so as to minimise the risk of predation [37]. Larger fields particularly if they provide high cover are safer for birds since they are less detectable [29,63]. The cover from cereal and weeds is a key factor for partridge survival [17,36,67]. However, larger cereal fields need to provide food and shelter. Potts [37] summarised the needs for incubating females with 1) the availability of dead vegetation as cover for nest building, 2) a given minimum cover height to protect the female, 3) the presence of field boundaries, and 4) the presence of linear structures to escape in the crops from predators. A first outcome of our modelling revealed that Vienna partridges during breeding not only selected for larger field size than in winter but preferred harvested fields, and at the same time avoided hedges, fallow land, meadows and greening (Fig 7). Typically, these structures are recorded to be a partridge magnet during breeding in large scale agricultural areas in order to ensure high vegetation cover as shelter and an insect-rich surrounding for the chicks. In our study, the avoidance indicates low habitat quality due to intensive management of meadows and fallow land with cutting times too early in summer during breeding and because old outgrown hedges may function rather as predator reservoirs. This is in line with Buner et al. [38] demonstrating that partridges used wild flower stripes in all seasons but avoided low quality hedges during breeding.

A second key outcome of our modelling process was that in winter the birds shifted habitat preference toward more risky habitats (Fig 6, see also [12]). Although less widely distributed in winter (Fig 3, but see [38]), the partridges preferred meadows but even human infrastructure reservoirs such as roads (Fig 6). Harmange et al. [12] discovered that in France over decades partridges got closer to predator risky structures such as roads. An explanation was the high number of released captive partridges for hunting purposes in the population that are not predator trained and lack in avoiding behaviour. In Vienna, the release of partridges may be ignored. We rather assume that in winter avoidance of predator reservoirs (woodlands and buildings) has weakened, and selection of human infrastructure (roads and tracks) that harbour seeds (see also [68,69]) has increased because partridges have a lack of choice to get access to scarce seed sources. In comparison to breeding time, the birds may better cope with predators in winter through increased group sizes and, thus, vigilance. Hence, they may afford to be more exposed in order to get access to food. Apart from group size, cover provided at this time of the year by vegetation besides roads and paths is further affecting vigilance and, hence, the time available for feeding [28].

In contrast to our findings, Buner et al. [38]) found a decrease in home range size from covey (15 ha) to breeding period (7 ha). Although we cannot provide a radio telemetry study to prove that home ranges were larger in winter, the birds were very concentrated in few areas (Fig 3). One explanation may be food concentration on specific fields and at pheasant feeding stations that provide continuous food availability and, hence, attracting and concentrating birds in specific areas and near roads. This is in line with Salek et al. 2004) demonstrating considerably unbalanced spatial arrangement with conspicuous local concentrations of grey partridges.

The deprivation of the grey partridge population in Vienna is an indicator for a loss of biodiversity in the farmland areas near the city. The partridge’s avoidance of meadows and greening during breeding may be an indicator of direct partridge habitat alteration due to increasing landscape structuring and edge effect due to constructions of settlements and roads in the urban vicinity. This results in a loss in size and quality of partridge farmland habitats that is additionally altered by crop choice and pesticides reducing plant and insect food. Particularly high insect and seed abundance has been linked to higher densities of farmland birds, including grey partridge [36,37,70]). With declining breeding pairs, the grey partridge does not seem to adjust to these unsustainable landscape changes and farmland practices. Therefore, low food availability may explain the partridge’s large search areas for food and higher concentrations at sites which in turn might increase the bird’s susceptibility to predation and wet weather conditions.

The continuous drop in habitat quality caused by agricultural intensification may have increased the concentration of prey and the predation pressure on the few remaining suitable patches [22]. Low numbers in partridges increase further the risk of predation in winter since groups are smaller and, thus, group vigilance is reduced. After Kokko and Sutherland [71], low-density population may be further at risk as the nearly competition free situation increases the risk of the individuals to fall into ecological traps.

To improve the survival chances of the grey partridge, immediate efforts must be directed to peri-urban farmland protection and habitat improvement, including the reduction of pesticides and increasing surface area covered by large flower and fallow stripes in order to restore the density of cover, insects, and seeds in the landscape. A collateral predator management and stop of partridge hunting will ensure self-sustainable populations. The persistence of hunting activity, although with a limited effort, has probably contributed to the extinction of many sub-populations and is critically threatening the remaining ones [72]. Hence, even a very low rate of harvesting cannot be tolerated by the present continental populations. Only an integrated local management, involving hunters, farmers, municipalises and scientists can ensure the recovery of this species and, thus, its surrounding biodiversity.

## Supporting information

S1 Fig Map of study site showing position of study squares. (TIF)

S1 Table Locations of the centroids (longitude, latitude) of the 20 study squares. (CSV)

S2 Table Data from this study rough habitat scale. (CSV)

S3 Table Data from this study fine habitat scale. (CSV)

S1 Raw data (CSV)

S2 Raw data (CSV)
