# Supplementary figures and images for: Rural landscape dynamics over time and its consequences for habitat preference patterns of the grey partridge Perdix perdix
Source: PLoS One. 2021 Aug 19;16(8):e0255483. doi: 10.1371/journal.pone.0255483 (PMC8376057; doi:10.1371/journal.pone.0255483)

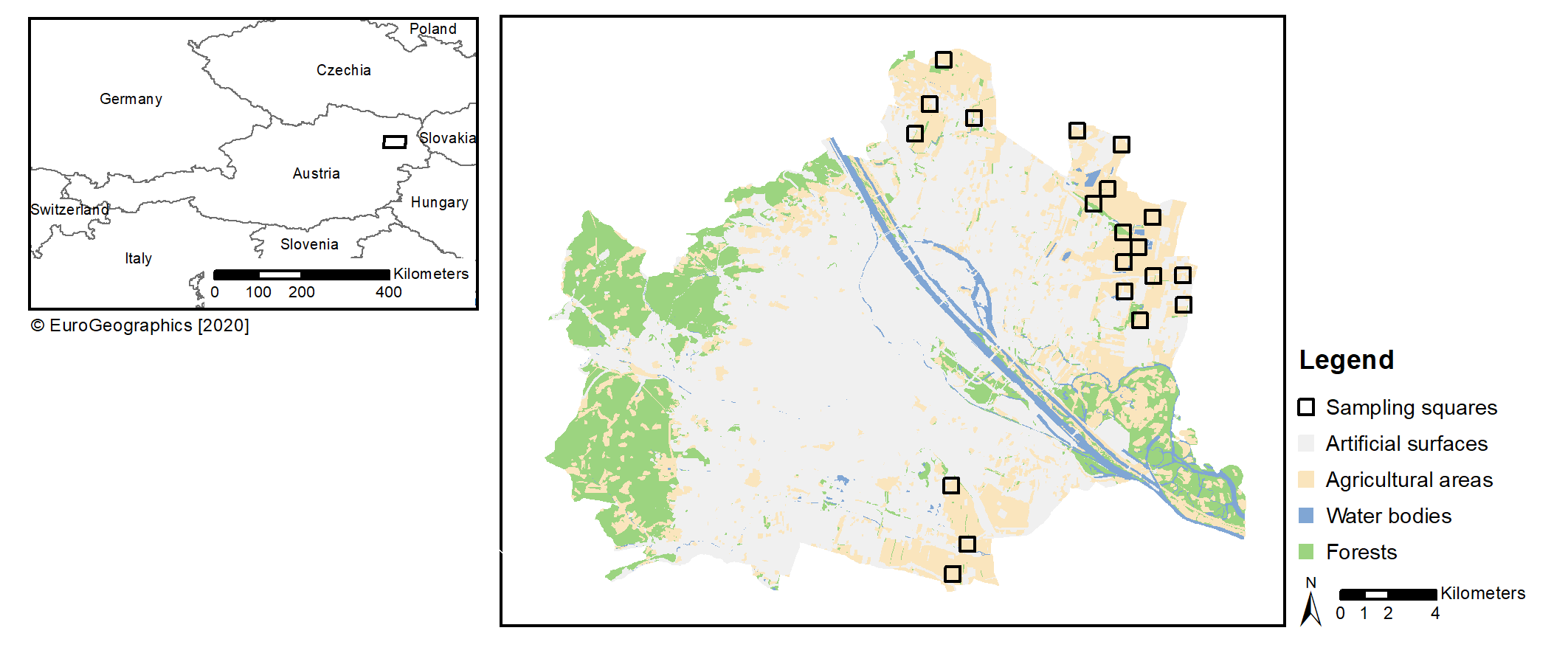

Supplement: S1 Fig — (TIF) [file pone.0255483.s001.tif]
